# The effect of climate change on laying dates, clutch size and productivity of Eurasian Coots *Fulica atra*

**DOI:** 10.1007/s00484-020-01972-3

**Published:** 2020-09-17

**Authors:** Lucyna Halupka, Beata Czyż, Carlos Moises Macias Dominguez

**Affiliations:** 1 Ornithological Station, University of Wrocław, Sienkiewicza 21, 50-335 Wrocław, Poland; 2 Department of Behavioural Ecology, University of Wrocław, Sienkiewicza 21, 50-335 Wrocław, Poland

**Keywords:** Climate change, Breeding, Clutch size, Phenology, Eurasian Coot, *Fulica atra*

## Abstract

Climate change is affecting many living organisms; however, the responses of many of them remain unknown. In this paper, we present the results regarding the response of a bird species from the rallid family to the increased temperatures during the breeding season. We analysed the breeding data of Eurasian Coots nesting during 30 seasons between 1972 and 2019. During the study period, mean temperatures in April, the month when Coots start nesting, increased by 3.5 °C, and in months corresponding with the species breeding season by 2.6 °C. Breeding Coots advanced their earliest and median laying dates across the study period; however, the duration of their breeding season remained unchanged. We did not detect any significant temporal changes in clutch size, but clutches have become much more variable in size throughout the study period. Nest failures and production of offspring per nest did not change over the study period; however, the production of young per successful nest significantly declined. It is likely that this decline is the effect of mismatch between the period of food abundance (dipterans collected from water), and hatchling emergence, which is advanced due to change in climate. Future studies investigating the occurrence of dipteran resources at water bodies are needed to test this hypothesis.

**Electronic supplementary material:**

The online version of this article (10.1007/s00484-020-01972-3) contains supplementary material, which is available to authorized users.

## Introduction

The global mean temperature has been steadily rising across the last decades, and it seems that the current warming trend is unprecedented over decades to millennia (IPCC 2014; Houghton 2015). Changes in temperature affect other meteorological conditions, like humidity and precipitation patterns, and result in a change of climatic conditions in many places of the globe. Climatic changes affect a wide range of living organisms, including birds. Although birds remain one of the best studied groups with regard to climate change, the proportion of species investigated in this context is still very low. Most avian studies related to climate-driven changes in breeding performance have concentrated on passerines (Husby et al. 2009; Vatka et al. 2011; Tarwater and Arcese 2018; Dyrcz and Czyż 2018), birds of prey and owls (Lehikoinen et al. 2011; Terraube et al. 2014), waterbirds (Moe et al. 2009; Wanless et al. 2009; Nolet et al. 2020) and waders (Smart and Gill 2003; Torti and Dunn 2005), while other groups of birds have been studied extremely rarely. Furthermore, the vast majority of studies have investigated the effect of rising temperatures on arrival and migratory dates, while the impact of climate change on population productivity and other fitness-related measures remains largely unknown.

The most clear pattern emerging from previous avian studies is that many birds advance laying dates in response to climate change (Dyrcz and Czyż 2018; Dunn 2019; Radchuk et al. 2019). The second frequently studied trait has been clutch size. In most studied species, earlier laying has not been associated with producing larger clutches (Dyrcz and Halupka 2009; Lehikoinen et al. 2011; Dunn and Møller 2014), though in some cases advanced breeding has led to increased clutch size and higher breeding success (Schaefer et al. 2006; Dunn and Møller 2010). Changes in laying dates may also affect the duration of the entire breeding season, leading to its shortening or extension (reviewed by Halupka and Halupka 2017), and in turn may affect changes in offspring productivity. In particular, shortening of breeding season has been found in single-brooded species, frequently relying on single food resources (Drever and Clark 2007; Najmanová and Adamík 2009). In contrast, many double-brooded species have extended their seasons in response to climate change. They often use multiple food resources and may take advantage of a longer vegetative season, lay more clutches and hence produce more offspring annually (Halupka et al. 2008; Bulluck et al. 2013). Breeding productivity may also decrease, even if the length of breeding season remains unchanged, as a result of a mismatch between the time of peak of food resources and the time the chicks are fed by the parents (Visser et al. 2004; Drever and Clark 2007; Reed et al. 2013, but see Cresswell and McCleery 2003; Vatka et al. 2011)*.* On the other hand, higher temperature, precipitation or humidity may be associated with higher productivity of ecosystems, and higher availability of food resources (Avery and Krebs 1984; Thomas et al. 2017). Higher temperatures may also result in important changes within the existing food chains, and affect the functioning of the whole ecosystems. For example, the shrinkage of sea-ice used for hunting by polar bears forces them to hunt on alternative terrestrial food, the eggs of colonial, ground-nesting birds (Prop et al. 2015). Similar shifts in predation pressures may be found in other ecosystems as a result of changes in vegetation cover, the rate of plant growth, altered phenology of insects, etc. (Dunn and Møller 2019).

The Eurasian Coot *Fulica atra* is a representative of the rail and crake bird family, the Rallidae. It breeds on freshwater lakes and ponds across the majority of Europe, Asia, Australia, and parts of Africa. Birds nesting in colder regions migrate further south and west in winter as the waters freeze, but populations in milder climates remain resident. Nests of the species have a form of platforms built of dead reeds and grasses, placed near the water’s edge or on structures protruding from the water. Clutches usually contain 5–10 eggs (Cramp et al. 1980). The young leave the nest soon after hatching, but are dependent on adults for food during first weeks. In central Europe, Coots lay eggs from April to July, while in the south-west even from February to September.

In this paper, we analyse changes of breeding parameters of Eurasian Coots nesting in Poland between 1972 and 2019. The goal of this project was to find out whether climatic changes have affected laying dates, clutch size and offspring productivity.

## Materials and methods

### Breeding data

**Fig. 1:**
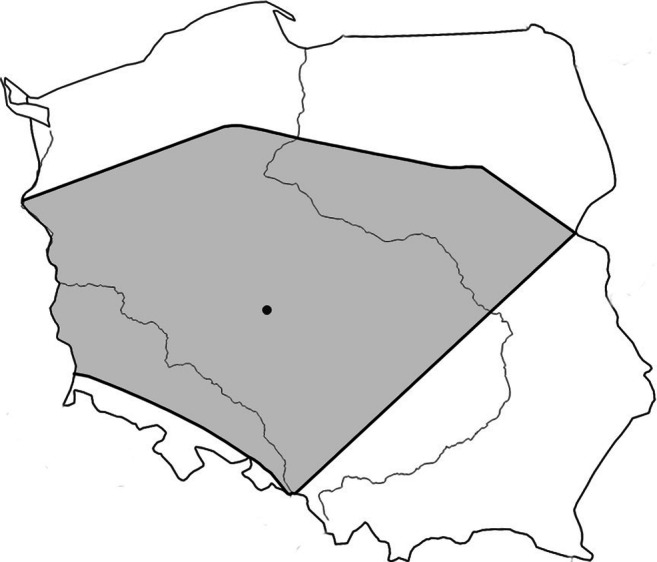
Study area, the bioclimatic region IV “Central” in Poland (following Blazejczyk 2004), is marked in grey. The location of the city of Kalisz is shown as a black dot

Breeding data of Coots nesting in Poland were obtained from the Polish nest-card scheme located at the University of Wrocław. The cards contained the information about more than 3000 nests of the species recorded since 1972 within the whole area of Poland. However, as climatic conditions in different parts of Poland differ considerably (8 bioclimatic regions are distinguished; Blazejczyk 2004), differences in the annual share of records coming from areas with different climates might have biased the data. To avoid this, we included only the data from one, the largest Polish bioclimatic region (Blazejczyk 2004) comprising areas situated in central and western Poland (Fig. 1). For this reason, we excluded data from 995 cards collected in other bioclimatic regions. Furthermore, we excluded years with fewer than 10 nests for all the analyses related to laying dates (following Halupka and Halupka 2017) and with fewer than 5 nests in other analyses (following Torti and Dunn 2005; Møller et al. 2010). Finally, data on 1794 nests of the species observed during 30 breeding seasons, between 1972 and 2019, were included (data from years 1973–75, 1977, 2002–2003, 2005–2011 and 2012–2016 were not available for any analysis due to very low sample sizes). As not all the analysed data were available on each nest-card, sample sizes differed between the analyses. All variables related to laying dates have been calculated using the data from 1590 nests (27 seasons between 1972 and 2012), on clutch size—from 1214 nests (30 seasons, 1972–2019), and on nest success and young production on 477 nests (26 seasons between 1978 and 2019).

To estimate the annual clutch size, we assumed that complete clutches contained at least 5 eggs (c.f. Cramp et al. 1980; Rizi et al. 1999). If a nest was found during egg laying, we estimated the first-egg laying dates by backdating, assuming one egg was laid per day. If the nest with a complete clutch was found and it did not survive until hatching, we also backdated the laying date assuming the incubation lasts for 24 days (Lelek 1958) and the nest visit(s) occurred in mid incubation, minimising the estimation bias (Mayfield 1975; Wesołowski and Mokwa 2013). For some nests (where the drawings were provided by observers), the incubation stage was determined by immersion method (c.f. Weidinger 2001). Hatching dates were determined accurately (if observed), or estimated assuming that hatching of the first young occurred 24 days after laying of the first egg (Lelek 1958; Cramp et al. 1980).

As nest card data are subject to bias introduced by variable sampling efforts (Weidinger 2001), for each year we estimated 5th and 95th percentiles of the first-egg dates, following other authors analysing laying trends from nest-card records (e.g. Najmanová and Adamík 2009). Likewise, the interval between these dates was an estimation of the season length (c.f. Torti and Dunn 2005; Møller et al. 2010).

To assess the variability of clutch sizes, we calculated within-clutch coefficient of variation following the formula:

$$ C\mathrm{V}=\frac{s\cdotp 100}{\overline{\Upsilon}} $$

where *s* is the standard deviation of clutch size in a year and $\overline{\Upsilon}$ is the mean clutch size. To avoid bias of the calculated coefficient of variation due to small sample sizes in some study years, we applied the correction (Sokal and Rohlf 1995):

$$ {C\mathrm{V}}^{adj}=\left(1+\frac{1}{4n}\right)\cdotp CV $$

where CV^*adj*^ is the bias-adjusted coefficient of variation and *n* is clutch size.

A nest was defined as successful if it produced at least one young (fledgling), and the annual nest success was expressed as the proportion of successful nests. For each study year, we also calculated the production of young per nest (the mean number of young that successfully left an average nest, calculated by dividing the total number of young leaving all nests by the number of nests with known outcome) and the production of young per successful nest (the mean number of young that left a successful nest in a year), following other authors (e.g. Schaefer et al. 2006; Dyrcz and Czyż 2018).

### Meteorological data

Weather data were obtained online from TuTiempo Network (https://en.tutiempo.net/climate) for the city of Kalisz (TuTiempo 2020), located in the centre of the study area (51.78 N, 18.08 E). We used mean diurnal temperatures (in Celsius degrees) and average relative humidity (in %) for months corresponding with the species breeding season (April–July). Similar data were obtained for precipitation (monthly sums in mm).

### Statistical analysis

To study the changes in the timing of breeding, we analysed (1) the earliest laying date observed in a season; (2) the median laying date calculated for all nests in the season, including second broods as well as re-nests after previous nest failure; and (3) the latest laying date and (4) the length of the laying period (the time between the date of laying of the first egg in the earliest and the latest nest in a season). The analysis of reproductive parameters included clutch size, nest success (i.e. the proportion of nests that produced at least one hatchling) and mean production of young per nest and per successful nest.

In the analyses of changes in breeding parameters over the study period (1972–2019), we used Mann-Kendall trend test corrected for serial dependence using the R package modifiedmk (Patakamuri and O'Brien 2019). Sen’s slope estimator was used for computing the changes in mean temperatures, humidity and breeding season advancement across the study years. We used partial Mann-Kendall test (Libiseller and Grimvall 2002) to analyse whether the detected trends were caused by significant trends in the temperatures and humidity. The analysis was done with the R package trend (Pohlert 2018) and different mean temperatures and humidity were used depending on a tested breeding parameter (see Table 1). We also calculated Kendall correlation coefficients between the breeding parameters with a significant trend over time and meteorological data. *P* values of multiple tests were corrected for multiple comparisons using Holm method (Holm 1979). Statistical analyses were performed using R (version 3.6.3) software (R Development Core Team 2020).

**Table 1:** Values of partial Mann-Kendall test statistic for relationships between significant trends in Coot breeding parameters and significant trends in climatic variables

Breeding parameter	April/April–July temperature (z, p)	April/April–July humidity
Earliest laying date	− 2.86, 0.024	− 3.56, 0.004
Median laying date	− 2.59, 0.028	− 3.56, 0.004
Median hatching date	− 2.62, 0.028	− 3.56, 0.004
Clutch CV^adj^	2.70, 0.028	3.42, 0.004
Production per successful nest	− 1.59, 0.113	− 2.80, 0.025

Earliest laying date trend was tested for April temperature/humidity trends while the rest of the breeding parameters were tested for seasonal (April–July) climatic data trends. *P* values were adjusted for multiple comparisons using Holm method

## Results

### Changes in meteorological data

**Fig. 2:**
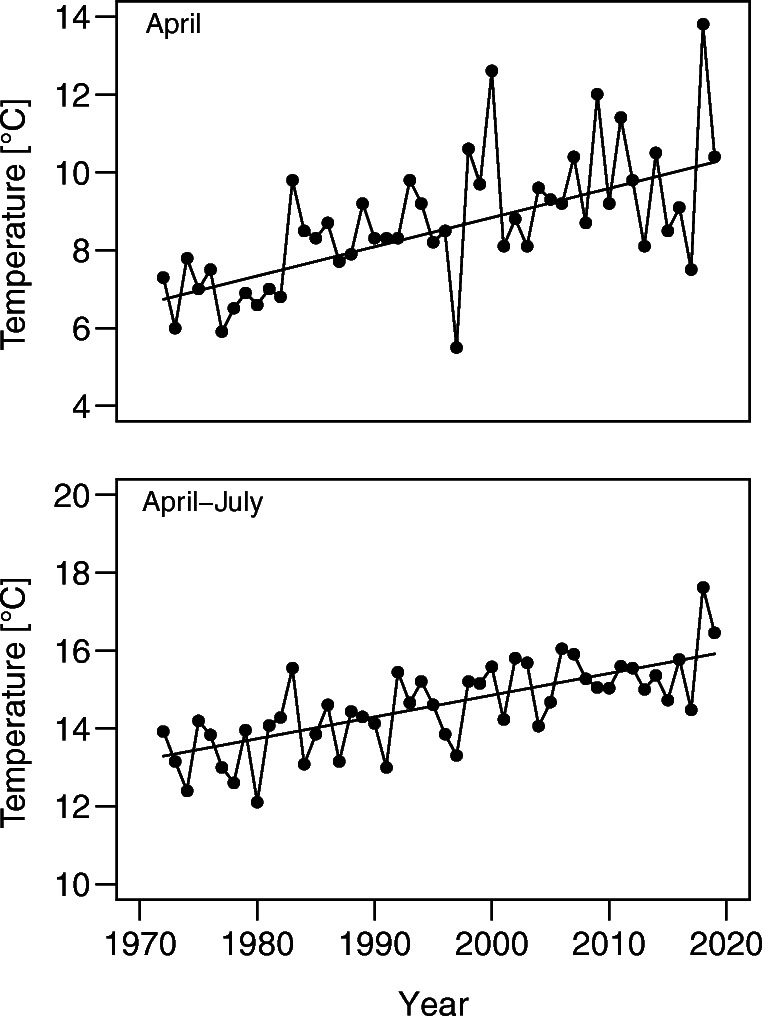
Mean temperatures of April and April–July in central Poland between 1972 and 2019

Mean monthly temperatures in April, May, June and July increased significantly across the study period (Mann-Kendall test, April: τ = 0.46, *p* = 0.001, May: τ = 0.21, *p* = 0.036, June: τ = 0.31, *p* = 0.002, July: τ = 0.33, *p* < 0.001) and the trend for the whole breeding season was also significant (Mann-Kendall test, April–July: τ = 0.52, *p* < 0.001). The increase equalled 2.6 °C for the whole breeding season, and 3.5 °C for April, when Coots start nesting (Fig. 2). We have not detected any significant trend in precipitation levels, either for monthly totals or for the whole breeding season. Mean monthly humidity in April and July slightly decreased throughout the study period, but not in May and June (Mann-Kendall test, April: τ = − 0.22, *p* = 0.03, May: τ = − 0.14, *p* = 0.15, June: τ = − 0.14, *p* = 0.16, July: τ = − 0.28, *p* = 0.006), while the trend for the whole breeding season was also significant (Mann-Kendall test, April–July: τ = − 0.28, *p* = 0.005). The decrease in humidity equalled 6% for the whole breeding season, 7% for April and 8% for July.

### Changes in breeding parameters

**Fig. 3:**
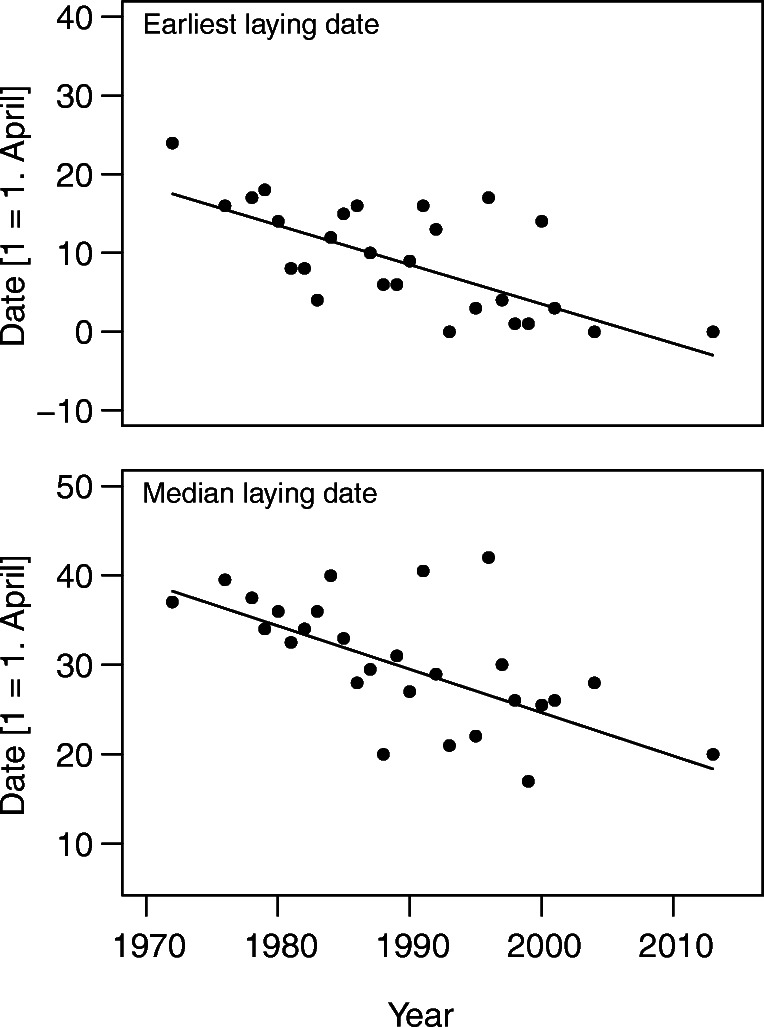
Changes in the earliest and median first-egg laying dates of the Eurasian Coots

During the study period, Eurasian Coots laid their eggs between 31 March and 14 July, but clutches started in July were very rare (4 cases, 0.22% of all nests). In different years, the first eggs in the earliest annual clutches were laid between 31 March and 24 April. Throughout the study period, Coots started laying their earliest clutches progressively earlier (Mann-Kendall test: τ = − 0.49, *p* < 0.001, *n* = 27); Fig. 3. Likewise, median annual laying dates have become significantly advanced by about 20 days (Mann-Kendall test: τ = − 0.48, p < 0.001, *n* = 27); Fig. 3. Median annual hatching dates advanced by about 18 days and the temporal trend was significant (Mann-Kendall test: τ = − 0.48, *p* < 0.001, *n* = 27). In contrast, the latest annual first-egg dates did not change significantly throughout the study period (Mann-Kendall test: τ = − 0.03, *p* = 0.85, *n* = 27). We did not find any significant changes in the duration of laying period (Mann-Kendall test: τ = 0.19, *p* = 0.17, *n* = 27). The trends in the timing of breeding (earliest and median laying dates and median hatching date) were significantly related to the trends in temperature and humidity, tested for April (earliest laying date) and April–July (median laying and hatching dates) temperature and humidity (Table 1). Median laying and median hatching dates were negatively correlated with seasonal (April–July) temperatures (respectively τ = − 0.43, *p* = 0.03, *n* = 27, and τ = − 0.40, *p* = 0.042, *n* = 27), while the earliest laying date was not significantly correlated to April meteorological data (all *p* > 0.05).

Complete clutches of Eurasian Coots usually contained seven eggs (range 5–14), and the mean annual values ranged 6.24–8.19. We have not detected any significant changes in clutch size throughout the study years (Mann-Kendall test: τ = 0.18, *p* = 0.261, *n* = 30). However, clutch sizes have become significantly more variable across the study period (Mann-Kendall test: CV^*adj*^: τ = 0.46, *p* = 0.006, *n* = 30). The trends in the coefficient of variation in clutch size were related to the trend in April–June temperature and humidity (Table 1). Nonetheless, we did not find the significant correlation between the coefficient of variation in clutch size and any of the seasonal (April–July) climatic data (all *p* > 0.05).

Nest success varied widely between years, but we did not find a significant temporal trend (Mann-Kendall test: τ = 0.08, *p* = 0.591, *n* = 26). As a result, the production of young per nest also did not show any significant trend across years (Mann-Kendall test: τ = 0.06, *p* = 0.674, *n* = 25). However, we found a negative significant trend in the production of young per successful nest (Mann-Kendall test: τ = − 0.41, *p* = 0.003, *n* = 26), which was significantly related to the trend in April–July humidity (Table 1). Nonetheless, no significant correlation between production of young per successful nest and any seasonal (April–July) meteorological variables was found (all *p* > 0.05).

## Discussion

In response to much higher temperatures during the breeding season, Eurasian Coots significantly advanced their laying dates. This is a result frequently observed in other bird species (Halupka et al. 2008; Vatka et al. 2011; Dyrcz and Czyż 2018; Smith et al. 2020) but not all (Nielsen and Møller 2006; Wright et al. 2009).

Because the earliest laying dates have advanced and we have not found any significant changes in the latest laying dates, this should result in the lengthening of the laying period of the species (Halupka and Halupka 2017). However, we did not observe the extension of the laying period (and hence the whole breeding season) throughout the study period. It seems that the cessation of the breeding season of Eurasian Coots was quite variable among years, and hence no consistent pattern in the season length has been detected (see Dyrcz and Halupka 2009). This result contradicts the assumption that multi-brooded species with a long breeding season, like the Coot (Cramp et al. 1980), should take advantage of a longer growing season and extend their breeding seasons (Dunn and Winkler 2010). However, although Coots are able to re-nest or even raise second broods and their season is quite long (Cramp et al. 1980), they do not belong to typical multi-brooded species, regularly raising two or three clutches annually (Dhondt 2010). Furthermore, earlier studies performed in the species have revealed a convex seasonal pattern in the production of young per brood. The experiments confirmed a causal relationship between laying date and fledging success, and experimental delay in the timing of breeding in the second half of the season resulted in a decline of breeding success (Brinkhof et al. 1993). These results suggest that Eurasian Coots may be strongly dependant on the optimal, short-term food resources for their young (Horsfall 1984), and hence may not benefit from the prolongation of the breeding season.

This study is another one to reveal that clutch size did not increase with the advancement of laying dates. Some papers on climate change hypothesised that in species in which the laying date is correlated with clutch size, advanced breeding may result in an increased number of eggs (Dunn and Winkler 2010). This was indeed found in some species (e.g. Schaefer et al. 2006), but not in the majority of studied ones (Dunn and Møller 2014). However, we found an interesting trend, showing that the coefficient of clutch variation increased in time, indicating that clutches have become more variable in size in recent years. This may indirectly suggest that foraging conditions for breeding females have become more variable across the breeding season in recent years.

We found a negative trend in the production of young per successful nest though clutch size did not change significantly across years. This suggests that partial losses increased throughout the study period. The most likely these losses occurred soon after hatching: the number of hatched eggs was usually estimated based on the number of young observed with adults soon after hatching. First 2 weeks after hatching and nest leaving are critical for survival of young Coots, and many of them die during this period (Horsfall 1984; Brinkhof et al. 1993). Our analysis suggests that mortality of hatchlings may have increased throughout the study period; however, the underlying mechanisms remain unclear. One possible mechanism is associated with a mismatch between the occurrence of food for the young and the time when young need most food (Visser et al. 2004; Thomas et al. 2001). During 2 weeks after hatching, young Coots are fed by their parents predominantly with dipterans picked up from the water surface, and this time period is critical for their survival (Horsfall 1984). The dipterans are characterised by a convex seasonal pattern of abundance (Horsfall 1984). Previous experimental studies revealed that the young hatched before and after the peak of dipteran abundance suffered lower survival, suggesting that proper timing is crucial for survival of young Coots (Brinkhof et al. 1993). More than 25 years ago, when these experiments were conducted, most young hatched during the peak of dipteran prey. It is very likely that today these two phenological events are not synchronised: the development of invertebrates is strongly temperature-dependant (Regniere et al. 2012; Esbjerg and Sigsgaard 2019), and the increase in spring temperatures has been very strong in the study area. Furthermore, many studies suggest that advancements in insect phenology are stronger than the ones of birds (e.g. Polgar et al. 2013). Therefore, it is likely that nowadays the peak of dipteran abundance is advanced in relation to the Coot phenology, resulting in food shortage for the Coot young and their declined survival. Such unequal phenological shifts at different ecological levels resulting in lower offspring production have been reported for several other species (e.g. Drever and Clark 2007; Van der Jeugd et al. 2009; Donelly et al. 2011; Dunn et al. 2011; Te Marvelde et al. 2011) and probably are more widespread than we supposed. The mismatch hypothesis seems to explain the increased mortality of young Coots; however, at present, we have no data to estimate shifts in diptera abundance in pond ecosystems, so we cannot test this hypothesis. It is also possible that the mortality of young Coots has different reasons (e.g. higher predation rate from the new, introduced predators, e.g. the American mink, changing water levels) or several factors are involved.

## Conclusions

Climate change has strongly affected the reproductive biology of Eurasian Coots. The birds started nesting significantly earlier but their breeding season length remained unchanged. Although the clutch size and nest success have not changed across years, the productivity of successful nests has significantly declined, probably as a result of mismatch between the occurrence of dipterans in water (the main food for young Coots) and the Coot breeding phenology. Future studies are needed to better understand the mechanisms explaining partial losses in the Coot offspring.

## Electronic supplementary material

ESM 1 (XLSX 10 kb)
